# A phase 1 experimental medicine study of anti-CD3 monoclonal antibody in rheumatoid arthritis

**DOI:** 10.1093/immadv/ltaf034

**Published:** 2025-12-03

**Authors:** Catherine A Lawson, Rachel Harry, Bridget Griffiths, Geoff Hale, Herman Waldmann, John D Isaacs

**Affiliations:** Academic Unit of Musculoskeletal Medicine, University of Leeds, Leeds, United Kingdom; Molecular Medicine Unit, Clinical Sciences Building, St. James’s University Hospital, Leeds, United Kingdom; Translational and Clinical Research Institute, Newcastle University, Newcastle upon Tyne, United Kingdom; Academic Unit of Musculoskeletal Medicine, University of Leeds, Leeds, United Kingdom; Musculoskeletal Unit, Newcastle upon Tyne Hospitals NHS Foundation Trust, Newcastle upon Tyne, United Kingdom; Sir William Dunn School of Pathology, University of Oxford, Oxford, United Kingdom; Sir William Dunn School of Pathology, University of Oxford, Oxford, United Kingdom; Academic Unit of Musculoskeletal Medicine, University of Leeds, Leeds, United Kingdom; Molecular Medicine Unit, Clinical Sciences Building, St. James’s University Hospital, Leeds, United Kingdom; Translational and Clinical Research Institute, Newcastle University, Newcastle upon Tyne, United Kingdom; Musculoskeletal Unit, Newcastle upon Tyne Hospitals NHS Foundation Trust, Newcastle upon Tyne, United Kingdom; NIHR Newcastle Biomedical Research Centre at Newcastle upon Tyne Hospitals and Newcastle University, Newcastle upon Tyne, United Kingdom

**Keywords:** therapeutic tolerance, immune modulation, immunotherapy, rheumatoid arthritis, otelixizumab, anti-CD3

## Abstract

**Introduction:**

An Fc-mutated chimeric aglycosyl anti-CD3 monoclonal antibody (mAb), otelixizumab, has been used successfully to treat renal transplant rejection and type 1 diabetes, with reduced toxicity compared with traditional anti-CD3 therapies such as OKT3. The aim of this study was to seek preliminary safety data for otelixizumab in rheumatoid arthritis (RA).

**Methods:**

A small Phase 1 experimental medicine study was performed in six participants with RA. The primary outcome measure was safety, with a focus on first-dose cytokine release reactions and extent of CD3^+^ lymphopenia. Cytokine release was quantified using ELISA, and lymphocyte subsets by flow cytometry. *In vitro* whole blood assays were used to interrogate the mechanisms underlying the first-dose cytokine release reaction. Clinical progress following therapy was monitored as an exploratory outcome.

**Results:**

All participants experienced a moderate first-dose cytokine release reaction. There was transient lymphopenia but no T-cell depletion, and a temporary CD8^+^ T-cell lymphocytosis occurred in all participants. In those who completed therapy, a sustained reduction in CRP following treatment was noted. In an *in vitro* whole blood assay, designed to mirror *in vivo* cytokine release, there was a trend for reduced cytokine production in seropositive RA compared with seronegative RA, psoriatic arthritis, or healthy controls.

**Conclusions:**

At the dosing regimen used, otelixizumab was associated with an unexpected and significant first-dose reaction in participants with RA.

## Graphical Abstract

**Graphical Abstract:**
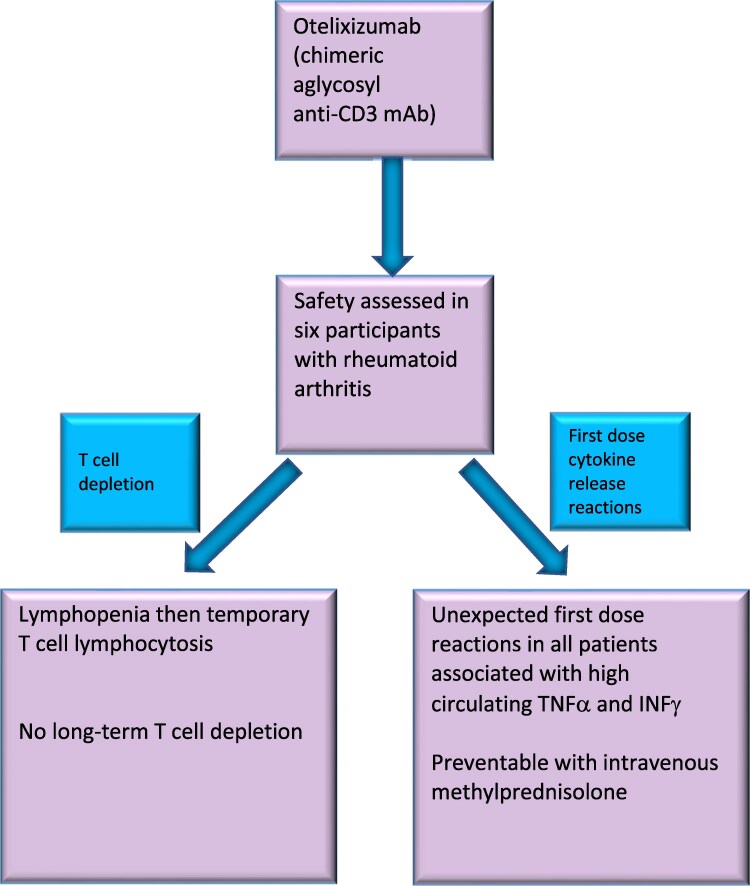

## Background

In animals, anti-CD3 monoclonal antibodies (mAbs) provide a potent tolerizing therapy as, for example, in models of Type 1 diabetes. Therapy results in reduction of effector T-cells and enhanced activity of regulatory T-cells, with concurrent reduction of target organ damage and long-term restoration of immune regulation [1]. In the clinic, an anti-CD3 mAb (OKT3) was used historically to reverse allograft rejection but its use was associated with significant toxicity, in particular a first-dose reaction. This was a consequence of cytokine release from targeted cells and could be life-threatening in the absence of adequate pretreatment [2–4]. The reaction could be reduced, but not eliminated, with glucocorticoid prophylaxis.

First-dose cytokine release reactions are a consequence of Fc gamma receptor (FcγR)-bearing accessory cells cross-linking anti-CD3 on the target cell leading to synchronized T-cell activation. A number of Fc-engineered mAbs have been designed to minimize FcγR interactions and reduce the likelihood of such reactions. These have been administered to participants with Type 1 diabetes, and various other immunopathologies, generally with good tolerance and significant therapeutic efficacy [5, 6].

Otelixizumab is an anti-human CD3 mAb, with a humanized heavy chain and a chimeric light chain [7]. The heavy chain has a single amino acid substitution at asparagine 297 which removes an N-linked glycosylation site that is critical for both complement activation and FcγR binding. The fully humanized antibody has been used to reverse renal allograft rejection episodes with minimal cytokine release [8]. In recent onset Type 1 diabetes, short-term therapy with otelixizumab was shown to preserve residual beta-cell function for at least 18 months [5]. Moderate flu-like symptoms were reported by the majority of participants on the first day of treatment, and immunogenicity was seen in 53% of patients. Benefits of therapy were still apparent after 48 months [9].

The current report describes a small phase 1 experimental medicine study of otelixizumab in participants with rheumatoid arthritis (RA), where there is a continuing unmet need for novel therapeutic approaches, particularly those that might harness tolerance-associated mechanisms.

## Methods and materials

### Chimeric aglycosyl anti-CD3 antibody

Otelixizumab was derived from two sources. Therapeutic otelixizumab was manufactured at the Therapeutic Antibody Centre (Oxford, UK) using standard methods [10] and formulated in phosphate-buffered saline. Otelixizumab used for whole blood assays was provided by GSK, Stevenage, UK.

### Study design and protocol

This was an open label, experimental medicine study on six participants with RA. It was approved by the Leeds (West) Local Research Ethics Committee, and all participants gave written informed consent.

Key inclusion criteria were: age 18–70; RA as defined by the 1987 revised ACR criteria [11]; duration of RA at least 5 years; previous failure of at least two synthetic DMARDs; active disease defined as DAS28 >5.1 plus three out of four of: six or more tender joints, six or more swollen joints, CRP >30 mg/dl and early morning stiffness >45 minutes. Exclusion criteria were chronic infection, previous treatment with biological therapy and previous malignancy.

The treatment protocol was modelled on that used to treat renal allograft rejection [8] and Type 1 diabetes, with a target dose of 60–70 mg of mAb per participant over five consecutive days, administered by intravenous infusion. The precise regime was adapted with knowledge gained from each prior participant. Following treatment participants were assessed at regular intervals for 6 months. Longer term outcomes were obtained from routine clinical follow-up.

The primary outcome measure of the study was safety, with a focus on possible first-dose reactions and CD3^+^ lymphopenia [1].

### Serum cytokine quantification during first-dose reaction

Tumour necrosis factor alpha (TNFα) and interferon gamma (IFNγ) levels were measured in serum samples by ELISA (Diaclone, Besançon, France). Samples were taken at baseline, after 2 h, and at the end of mAb infusions. Additional samples were taken during first-dose reactions.

### Whole blood assays

To investigate factors influencing first-dose reactions with otelixizumab, *in vitro* whole blood assays were performed [12]. Samples were taken from individuals with active seropositive or seronegative RA, active psoriatic arthritis, or healthy controls (HC). Heparinized whole blood was incubated with otelixizumab at 1 or 10 μg/ml at 37°C for 1, 2, or 4 h. Lipopolysaccharide (LPS) 2 ng/ml (Sigma) was used as a positive control. Cytokine production from all antibody stimulated conditions was analysed using meso-scale discovery (MSD) technology (Rockville, Maryland, USA). This is a highly sensitive ELISA based multiplex assay capable of quantifying multiple analytes in parallel. Quantification of independent analytes is achieved using analyte specific detection antibodies coupled to MSD SULFO-TAG™, an electrochemiluminescent compound and a MSD Sector Imager. The 7-plex pro-inflammatory kit was used. This measured IL-1β, IL-12p70, IFNγ, IL-6, IL-8, IL-10, and TNFα. Cytokine production from the LPS conditions was analysed by ELISA (BD Pharmingen, Oxford, UK).

Statistical analysis was performed using a two-way ANOVA and a Bonferroni post-test to determine intergroup differences over time.

### Lymphocyte counts and lymphocyte subsets

Total lymphocyte counts and lymphocyte subsets were measured in the routine clinical immunology laboratory at St James’s University Hospital, Leeds. In flow cytometry assays, post-treatment circulating CD3^+^ T-cells were quantified using an antibody to an epitope on CD3 that did not overlap with otelixizumab.

## Results

Table 1 outlines the baseline characteristics for each participant in the study. All had highly active, refractory disease, previously treated with up to six anti-rheumatic drugs, with DAS28-CRP between 6.30 and 7.08 and CRP ranging from 27.9 to >213.0. This reflects the fact that this is a historical study that took place prior to the widespread availability of biologic therapy, with recruitment of participants from 1999–2002.

**Table 1.** Summary of participant characteristics at baseline

Participant (sex)	Age (years)	Disease duration (years)	Concomitant DMARD	Previous DMARDs	Rheumatoid factor	DAS28-CRP at baseline	CRP at baseline (mg/l)
1 (F)	50	5	Nil	SSZ, MTX, CIC	Positive	6.84	160.7
2 (M)	61	30	MTX	CIC, gold, PEN	Positive	6.88	83.0
3 (M)	67	6	Nil	SSZ, MTX, gold, PEN, HCQ, LEF, CYC	Positive	N/A	27.9
4 (F)	58	26	Nil	MTX, SSZ, HCQ, LEF, AZA, PEN, CIC	Positive	7.08	204.0
5 (F)	39	10	LEF	MTX, SSZ, gold, AZA, CIC	Negative	6.30	92.3
6 (F)	52	7	Nil	MTX, SSZ, HCQ, gold	Positive	6.48	213.0

SSZ, sulfasalazine; MTX, methotrexate; CIC, ciclosporin; PEN, penicillamine; HCQ, hydroxychloroquine; LEF, leflunomide; CYC, cyclophosphamide; AZA, azathioprine; N/A, not available.

### First-dose reactions

Most participants experienced a first-dose reaction of at least moderate severity. Typical symptoms included pyrexia, rigors, diarrhoea and vomiting, and hypotension. Table 2 summarizes adverse events including levels of circulating TNFα and IFNγ, and pre-medication used.

**Table 2.** Adverse events

Participant	Prophylaxis	Total (daily) dose of otelixizumab (mg)	Serum cytokines during reaction (pg/ml)		Infusion reaction	Additional adverse events
			TNFα	IFNγ		
1	Nil	70 (30/0/10/10/10/10)	2170	776	Day 1: Pyrexia 40.5°C, nausea and vomiting, diarrhoea, and hypotension	Cold sore (Day 5); pyrexia, rigors, diarrhoea, and vomiting (Day 13); flare of arthritis and lymphadenopathy (Week 4)
2	Nil	70 (10/10/10/10/30)	244	96	Day 1: Pyrexia 38.8°C, rigors, diarrhoea and vomiting, rash, and hypotension	Flare of arthritis, cold sore, and mouth ulcers (Day 8)
3	500 mg MEP (Day 1)	17 (10/7)	49 (Day 2)	86 (Day 2)	Day 2: Pyrexia 39.5°C, diarrhoea and vomiting, hypotension, and bronchospasm	None
4	100 mg p55TNFr-Ig	23 (3/10/10)	59	197	Day 1: Bronchospasm, rigors, and hypertension	Cardiac ischaemia (Day 3); mouth ulcers and rash (Week 2); sore throat and worsening joint pain (Week 4)
5a	250 mg MEP Day 1, 100 mg MEP Day 2, 50 mg MEP Days 3–5	60 (10/10/10/10/20)	187 (2 h into first dose)	81 (2 h into first dose)	None	Sore throat and mouth ulcers (Week 3)
5b	—	67 (7/10/10/10/30)	1911	757	Day 1: Pyrexia 38.5°C, nausea, rigors, and hypotension	Cold sores (Day 4)
6	150 mg MEP Day 1, 100 mg MEP Day 2	70 (10/0/10/10/10/30)	8	63	Day 1: Pyrexia 39°C, rigors, diarrhoea and vomiting	Oral candidiasis (Day 8); cutaneous vasculitis and arthritis flare (Week 3); large bowel vasculitis (Week 6)

MEP, methylprednisolone; p55TNFr-Ig, p55 tumour necrosis factor receptor-human IgG1 Fc fusion protein.

Based on previous experience in renal transplantation and Type 1 diabetes, participants one and two did not receive prophylaxis prior to otelixizumab. Participant one developed a significant infusion reaction after 30 mg otelixizumab (administered over 6 h), which included hypotension (Table 2); participant two received a smaller initial dose of 10 mg, administered over 8 h, but also developed a significant infusion reaction. All subsequent participants received eight hour infusions on Day 1. Participant three received prophylaxis with 500 mg methylprednisolone intravenously prior to otelixizumab infusion on Day 1. No infusion reaction occurred on Day 1 but a reaction occurred 5 h into the second infusion, which was administered without prophylaxis. This included bronchospasm and, due to concurrent chronic obstructive pulmonary disease, this participant was withdrawn from the study.

Because of data suggestive that TNF blockade could ameliorate CD3-induced cytokine release symptoms [13, 14], participant four received prophylaxis with a tumour necrosis factor-α antagonist (p55 tumour necrosis factor receptor-human IgG1 Fc fusion protein, also manufactured at the Therapeutic Antibody Centre) on Day 1. This did not prevent an infusion reaction, however, which comprised bronchospasm, rigors, and hypertension. Additionally, 8 h post-infusion on Day 3 the participant experienced an exacerbation of pre-existing angina pectoris and was withdrawn from the study.

Participant five received daily prophylaxis with methylprednisolone and did not experience an infusion reaction during 5 days of therapy. Due to a marked and sustained improvement in symptoms this participant requested and received a second course of treatment after 9 months (shown as Patient 5b in Table 2). There was no reaction following a 0.1 mg test dose of otelixizumab. An infusion of 10 mg was therefore commenced, without prophylaxis, but on this occasion the patient developed rigors, nausea, hypotension, and pyrexia after 90 min and only 7 mg otelixizumab were infused. Doses 2–5 were administered without further reactions.

Participant six developed pyrexia, rigors, diarrhoea, and vomiting on Day 1 despite prophylaxis with 150 mg methylprednisolone but continued to complete the course of treatment, with further methylprednisolone on Day 2.

### Other adverse events

All participants experienced a temporary worsening of joint symptoms 2–4 weeks after treatment. In some cases, new or more marked synovitis was present on examination. This was associated with a CD8^+^ T-cell lymphocytosis and, in some cases general malaise, sore throat, and lymphadenopathy. Serological testing for Epstein–Barr virus (EBV) reactivation was positive in Participant 1.

Other significant adverse events included oral candidiasis, mouth ulcers and cold sores (see Table 2). Participant six developed cutaneous vasculitis at Week 3 and diarrhoea at Week 6, with vasculitis confirmed on colonoscopic biopsy. However, there was a history of significant weight loss prior to study entry, and therefore, it was unclear whether this was present prior to receiving otelixizumab.

### Circulating cytokines

Cytokine levels during infusion reactions are reported in Table 2 and varied considerably between participants. We focussed on TNF**α** and IFN**γ** as these are the cytokines that have been clearly implicated in reactions to OKT3 [3]. TNF**α** serum levels reached more than 2 ng/ml in participant one but were scarcely detectable in Participant 6. Similarly, interferon-**γ** levels were high in participant one and low in Participant 6. Levels of both cytokines were high during the reaction experienced by Participant 2 during their second course of therapy. In general, there was a good correlation between levels of the two cytokines. The exception was participant four, in whom TNF**α** levels were disproportionately low, probably because of neutralization by p55TNFr-Ig pre-medication. This example reinforced a generally poor correlation between circulating levels of TNF**α** and IFN**γ** and clinical symptoms. Similarly, both cytokines were measurable during participant five’s initial course of therapy, which was not associated with an infusion reaction.

### Lymphocyte counts

**Figure 1.**
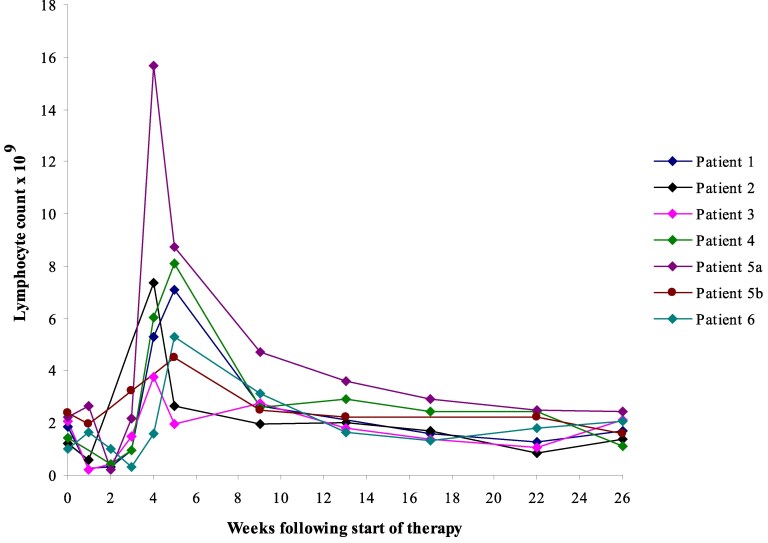
Changes in total lymphocyte counts for each participant. Total lymphocyte counts peaked between Weeks 4 and 6, and then returned towards baseline.

All participants developed lymphopenia immediately after therapy which was transient in all cases (Fig. 1). This was followed by a temporary CD8^+^ T-cell lymphocytosis around Weeks 3–6, even in participants who did not complete treatment. Similar observations in Type 1 diabetes were linked to a viral reactivation syndrome [5] and a regulatory T-cell expansion [15]. Lymphocyte counts subsequently returned to baseline values.

### C-reactive protein

**Figure 2.**
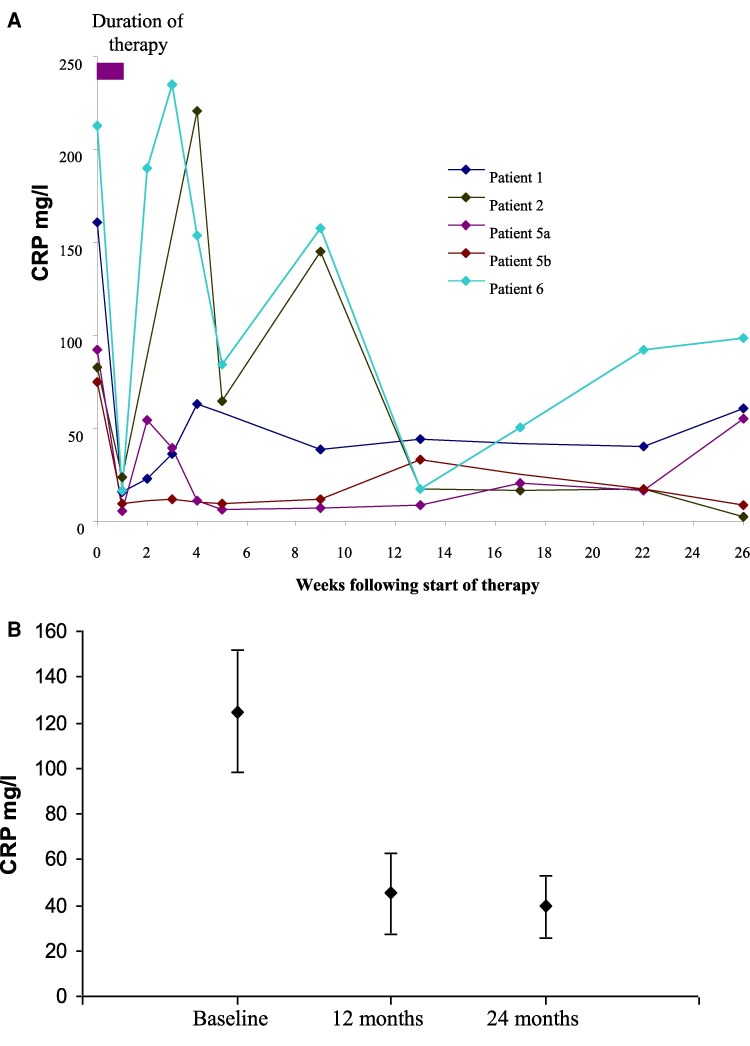
Changes in C-reactive protein levels following otelixizumab therapy. (a) shows CRP levels up to 6 months from the start of treatment for those participants who completed therapy. Participant six received oral prednisolone from Week 7. (b) Mean CRP levels at long-term follow-up for the four participants who completed therapy are shown.

CRP levels initially rose in association with infusion reactions and the viral reactivation syndrome. However, CRP levels were lower at 6 months than at baseline in all patients who completed therapy (Fig. 2a). Figure 2b demonstrates CRP values at long-term follow-up in participants who completed therapy.

### Whole blood cytokine release assays

**Figure 3.**
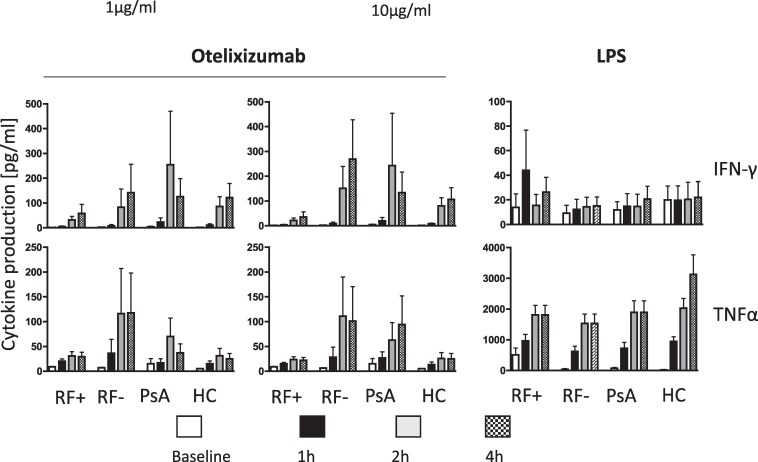
Whole blood assays using otelixizumab. Cytokine release in response to otelixizumab was determined in whole blood from individuals with seropositive (RF+) or seronegative (RF−) RA, individuals with psoriatic arthritis (PsA) and from HC. Whole blood was stimulated for up to 4 h with otelixizumab 1–10 µg/ml or with LPS 2 ng/ml as a positive control. Bars represent mean ± SEM of at least six individuals.

The development of significant cytokine release reactions in this study was unexpected from experience in renal transplantation and type 1 diabetes [5, 8]. Possible explanations for the more severe reactions seen in RA participants included systemic inflammation or possibly rheumatoid factor (RF). These were investigated using *in vitro* whole blood cytokine release assays using blood from individuals with active seropositive or seronegative RA, active psoriatic arthritis, and HC. Cytokine release was assessed following incubation with 1 and 10 μg/ml otelixizumab (Fig. 3).

There were no significant differences seen between the clinical groups, although there was a trend towards lower cytokine production using whole blood from individuals with seropositive RA compared with either seronegative RA or psoriatic arthritis.

### Participant vignettes

Four of six participants completed the course of treatment (Participants 1, 2, 5 and 6).

Participant 1 had a marked improvement in joint symptoms from Day 2 until Week 4. She then experienced an arthritis flare, in association with EBV reactivation, confirmed serologically, which settled spontaneously. After 4 months sulfasalazine was reintroduced for worsening of arthritis, and this was subsequently replaced by methotrexate. Both drugs had been previously ineffective but at follow-up 4 years after otelixizumab her disease was well controlled on methotrexate and low-dose prednisolone.

Participant 2 was taking methotrexate at baseline, having recently discontinued ciclosporin. He was commenced on sulfasalazine at Week 6 plus oral prednisolone due to increased joint symptoms. Sulfasalazine was stopped at Month 7 when he was in clinical remission. His disease remained well controlled after 4 years taking methotrexate and 2.5 mg prednisolone.

Participant 5 was taking leflunomide at baseline. Ten days prior to trial entry she had received two pulses of IV methylprednisolone with no effect on disease activity. Her joint symptoms and fatigue started to improve from Day 2 of otelixizumab therapy. She received an intra-articular wrist injection at Month 4 with no change in systemic treatment. She reported feeling as well as she had ever felt since her arthritis onset, with greatly improved quality of life. However, joint pain and morning stiffness started to return by Month 6 and she was retreated after 9 months. The second course of treatment led to improvement for 3 months following which her synovitis returned.

Participant six presented with systemically active disease (DAS28-CRP 6.48, CRP 213) and weight loss. Leflunomide treatment was stopped because of the latter. This participant did not experience improvement of joint symptoms with therapy and had a complicated course. On Day 20, she developed a purpuric rash on her fingers and petechial lesions on her calves and feet, in association with worsening synovitis. She developed a necrotic finger lesion and received IV methylprednisolone plus iloprost. Persistent diarrhoea started on Day 43 and a colonoscopic biopsy indicated vasculitis, which responded to oral prednisolone and mesalazine. Her arthritis remained active despite prednisolone.

## Discussion

We performed a small Phase 1 experimental medicine study of an Fc-mutated anti-CD3 mAb, otelixizumab, in RA. This historical study was performed prior to widespread availability of biologic therapy, and our participants had highly active, DMARD-refractory disease. Most participants experienced a significant first-dose reaction, which was unexpected according to data available at the time from subjects receiving otelixizumab for allograft rejection and Type 1 diabetes. As with OKT3, the reaction was accompanied by high levels of circulating TNFα and IFNγ [3]. The reaction appeared preventable with methylprednisolone at a dose of between 150 mg (ineffective in Participant 6) and 250 mg (effective in Participant 5a), which accords with historical data using OKT3 [16]. The lack of prevention with p55TNFr-Ig, which appeared to neutralize circulating TNFα, is in keeping with murine data demonstrating that, in contrast to anti-TNF monoclonal antibodies, soluble TNF receptor does not prevent anti-CD3 triggered cytokine upregulation. The efficacy of anti-TNF antibodies appears to reflect binding and cross-linking of membrane TNF expressed on the surface of activated T lymphocytes, inhibiting the transcription of both IFNγ and IL-6 [17, 18].

First-dose reactions are seen with a number of cell-binding therapeutic mAbs. OKT3 is the classical example, with symptoms such as fever, chills, nausea and vomiting, headache, bronchospasm, and hypotension occurring after 2 h of treatment [16]. RA participants exhibited a first-dose reaction when treated with the lymphocytotoxic mAb alemtuzumab (CAMPATH-1H) comprising chills, fever, nausea, and rash [19]. A similar reaction occurs in RA patients treated with rituximab and is again preventable by glucocorticoid prophylaxis [20]. The most infamous example of mAb-induced T-cell activation occurred with TGN-1412, with sequelae that included renal failure, lung injury and disseminated intravascular coagulation [21].

*In vitro* studies with alemtuzumab suggested that cross-linking of cell-bound mAb by CD16 (FcγRIIIa) on NK cells led to cytokine release from the NK cells themselves [22]. In contrast, cytokines released with OKT3 originate from the targeted lymphocytes, as a consequence of mAb-induced cross-linking of CD3 and cell activation [23]. The Fc engineering of otelixizumab was designed to reduce cytokine release by minimizing interactions with FcγRs. In patients with Type 1 diabetes, administration of 24 mg, without glucocorticoid prophylaxis, was associated with severe headache and vomiting; a reduced dose of 8 mg was associated with gastrointestinal symptoms, fever and headache, but not of sufficient severity to require prophylaxis; median peak TNF-α levels were 527 pg/ml at 1 h [5]. This is within the range measured in our study although nanogram levels were measured in two participants (Table 2). Therefore the reactions experienced in RA may reflect either a heightened propensity to cytokine release or heightened sensitivity to circulating cytokines, perhaps reflecting significant levels of background inflammation (Table 1).

Our *in vitro* whole blood assays exploring otelixizumab-induced cytokine release did not show significant differences between the clinical groups analysed. However, there was a trend towards higher cytokine release using whole blood from individuals with seronegative RA and psoriatic arthritis, compared with individuals with seropositive RA. We had initially considered that RF might enhance cytokine release because it has been shown to bind aglycosyl IgG Fc regions *in vitro*, and IgG RF could therefore cross-link therapeutic mAb at the T-cell surface [24]. Pentameric IgM RF could theoretically achieve the same without requiring FcγR interactions. However, our *in vitro* findings were not consistent with this hypothesis. Furthermore, without glucocorticoid prophylaxis Participant 5 with seronegative RA experienced an infusion reaction with high circulating cytokine levels, indicating that RF is not a prerequisite for a first-dose reaction. Indeed, our *in vitro* study suggests that RF may actually mitigate cytokine release. This observation is consistent with those of Jones *et al.* [25], who found that complement-dependent cytotoxicity mediated by rituximab was inhibited by IgM or IgA RF. Each of our participants had very severe RA with high levels of systemic inflammation, and it seems most likely that this enhanced their first-dose reactions, by sensitizing either CD3 T-cells or accessory cells (monocytes, NK cells). It may also be pertinent that RA is associated with high affinity FcγR polymorphisms, particularly individuals with seropositive, severe RA [26], and pharmacogenetic factors may have contributed. Nonetheless, despite its mutated Fc otelixizumab remains capable of T-cell activation, although the associated first-dose reactions, even in RA, are less severe than those seen with OKT3 [2].

The first-dose reactions associated with rituximab and alemtuzumab are associated with profound target cell depletion. This was not the case with otelixizumab (Fig. 1). An early and transient lymphopenia was observed in all subjects, likely explained by antigenic modulation as well as by marginalization of T-cells on endothelial cells that are activated upon the release of cytokines [1]. Subsequently, a lymphocytosis was observed some 2–3 weeks after therapy. Similar findings have been reported with otelixizumab in Type 1 diabetes, and attributed to viral reactivation with, for example, EBV [27]. We could only confirm EBV reactivation in one participant, but it is possible that alternative viruses contributed to this phenomenon.

Two participants (one and two) with previously refractory RA ultimately experienced prolonged benefit following treatment with otelixizumab. A third (Participant 5) had a good response lasting for more than 6 months. Participant 1 initially requested to leave the study after their first-dose reaction. However, 36 h later they experienced significant improvement in their refractory synovitis and requested continued treatment; a placebo response seems unlikely in this instance. Without a control group, it is not possible to formally assess efficacy but we have previously commented on apparent long-term modulation of disease with tolerogenic therapy in RA even when short-term outcomes have been disappointing [14]. Biomarkers that reflect the dysregulated immune modulation present in RA are required to adequately monitor the effects of such therapies; assessment of inflammation alone may prove wholly inadequate if tolerance takes several months to embed. Such biomarkers are also required to select appropriate doses of therapy to test in clinical trials [28].

Our work has highlighted the potential for disease-specific adverse reactions, expressly first-dose reactions, in response to a therapeutic mAb. We believe that systemic inflammation, perhaps in association with FcγR polymorphisms, may underpin our observations. This trial was performed with limited quantities of a biologic therapy manufactured in a licensed academic facility, and we based our dosing on extant data at the time. It was performed before the widespread availability of biologic drugs and the participants all had severe disease with limited therapeutic options. Anecdotal evidence of prolonged benefit in some participants could underpin further studies, with appropriate first-dose reaction prophylaxis and starting with significantly lower doses, as now required by regulators [29].

A study in collagen-induced arthritis, published subsequent to the work described here, supports the combination of anti-TNF and anti-CD3. In that work, consecutive treatment with anti-TNF and anti-CD3 monoclonal antibodies, but not with anti-CD3 alone, induced prolonged disease remission [30]. Furthermore, earlier work from the same team demonstrated a similar phenomenon whereby consecutive administration of anti-TNF and anti-CD4 provided therapeutic benefit not achieved with anti-CD4 alone [31]. These studies suggest the possibility of combining anti-TNF and otelixizumab in a future human study, mirroring our previous work with anti-TNF and anti-CD4 [14].

Our study emphasizes the importance of small experimental medicine studies before formal efficacy trials, particularly with therapeutic mAbs. It is rarely possible to extrapolate effects of mAbs from pre-clinical models or *ex vivo* assays into the clinic. This is particularly the case with cell-binding mAbs, which have repeatedly been shown to behave differently between species and *in vitro* [32]. Furthermore, biomarkers of immune dysregulation must be identified, and embedded within, studies of tolerogenic therapy, particularly in autoimmune disease where operational tolerance is hard to define. These will help to validate any suggestions of therapeutic benefit, including in randomized controlled trials.

## Conclusion

This experimental medicine study provides preliminary safety data around the use of otelixizumab for the treatment of RA. At the dosing regimen used, otelixizumab was associated with a significant first-dose reaction which was preventable with methylprednisolone. There was no T-cell depletion. Given anecdotal responses in some recipients, further studies to optimize both dosing and prophylaxis are warranted.

## Data Availability

The data underlying this article are available within the article.
